# Metabolic Origins and Transport of Vitamin E Biosynthetic Precursors

**DOI:** 10.3389/fpls.2017.01959

**Published:** 2017-11-14

**Authors:** Sébastien Pellaud, Laurent Mène-Saffrané

**Affiliations:** Department of Biology, University of Fribourg, Fribourg, Switzerland

**Keywords:** vitamin E, tocochromanols, mevalonate pathway, methyl erythritol phosphate pathway, homogentisate, prenyl pyrophosphate, Arabidopsis, nutrigenomics

## Abstract

Tocochromanols are organic compounds mostly produced by photosynthetic organisms that exhibit vitamin E activity in animals. They result from the condensation of homogentisate with four different polyprenyl side chains derived all from geranylgeranyl pyrophosphate. The core tocochromanol biosynthesis has been investigated in several photosynthetic organisms and is now well-characterized. In contrast, our current knowledge of the biosynthesis and transport of tocochromanol biosynthetic precursors is much more limited. While tocochromanol synthesis occurs in plastids, converging genetic data in Arabidopsis and soybean demonstrate that the synthesis of the polar precursor homogentisate is located in the cytoplasm. These data implies that tocochromanol synthesis involves several plastidic membrane transporter(s) that remain to be identified. In addition, the metabolic origin of the lipophilic isoprenoid precursor is not fully elucidated. While some genetic data exclusively attribute the synthesis of the prenyl component of tocochromanols to the plastidic methyl erythritol phosphate pathway, multiple lines of evidence provided by feeding experiments and metabolic engineering studies indicate that it might partially originate from the cytoplasmic mevalonate pathway. Although this question is still open, these data demonstrate the existence of membrane transporter(s) capable of importing cytosolic polyprenyl pyrophosphate such as farnesyl pyrophosphate into plastids. Since the availability of both homogentisate and polyprenyl pyrophosphates are currently accepted as major mechanisms controlling the type and amount of tocochromanols produced in plant tissues, we summarized our current knowledge and research gaps concerning the biosynthesis, metabolic origins and transport of tocochromanol biosynthetic precursors in plant cells.

## Introduction

**FIGURE 1:**
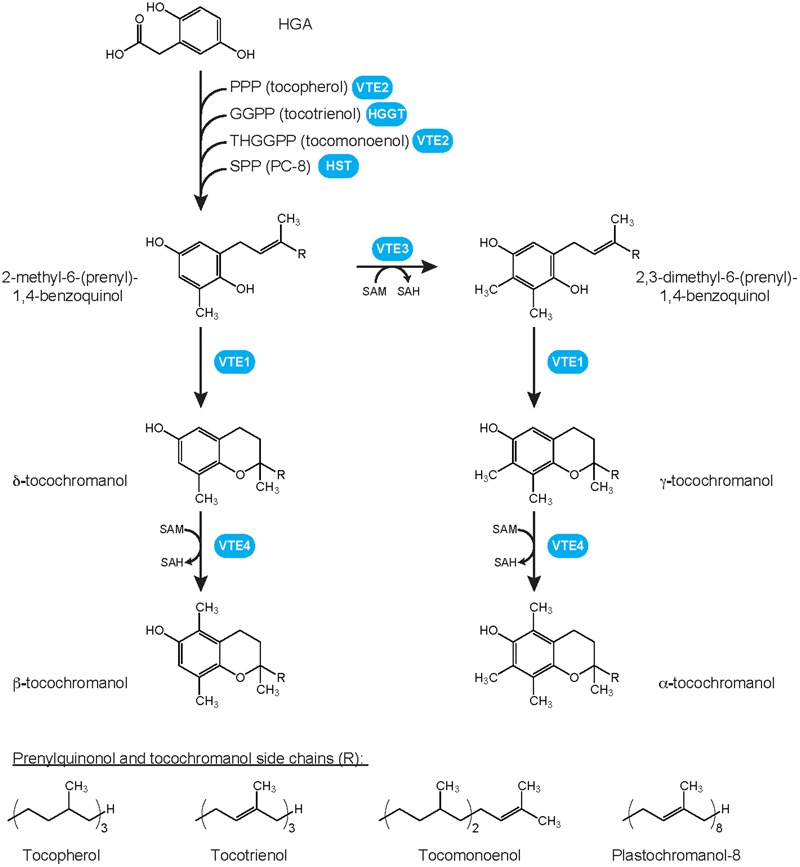
Plant tocochromanol biosynthetic pathways and chemical structures of prenylquinols and tocochromanols. Whereas all four methylated forms, i.e., α-, β-, γ- and δ-tocochromanol, have been identified in wild-type plants for tocopherols, tocotrienols, and tocomonoenols, only the γ- form of solanesyl-derived tocochromanol PC-8 has been identified in wild-type plants. Its methylated form, methyl PC-8, has been found only in transgenic plants overexpressing *VTE4.* GGPP, geranylgeranyl pyrophosphate; HGA, homogentisate; HGGT, HGA geranylgeranyl transferase; HST, HGA solanesyl transferase; PC-8, plastochromanol-8; PPP, phytyl pyrophosphate; SAH, *S*-adenosylhomocystein; SAM, *S*-adenosylmethionine; SPP, solanesyl pyrophosphate; THGGPP, tetrahydrogeranylgeranyl pyrophosphate; VTE, vitamin E biosynthetic enzyme.

Tocochromanols are amphipathic compounds typified by a chromanol ring (**Figure 1**). To date, four types of tocochromanols, namely tocopherols, tocotrienols, plastochromanol-8 (PC-8) and tocomonoenols, have been identified in higher plants. These organic compounds drew the attention because tocopherols and tocotrienols exhibit vitamin E activity that is notably essential for animal reproduction (Evans and Bishop, 1922). Tocochromanols are mostly produced by photosynthetic organisms such as plants, algae and some cyanobacteria, and by the non-photosynthetic parasite causing malaria *Plasmodium falciparum* (Sussmann et al., 2011; Mène-Saffrané and Pellaud, 2017). Tocochromanol biosynthesis is initiated by the condensation of two biosynthetic precursors, the polar homogentisate (HGA) and a lipophilic polyprenyl pyrophosphate that varies according to the type of tocochromanol. The polyprenyl precursor of tocopherols is phytyl pyrophosphate (PPP), geranylgeranyl pyrophosphate (GGPP) for tocotrienols, solanesyl pyrophosphate (SPP) for PC-8, and tetrahydrogeranylgeranyl pyrophosphate (THGGPP) for tocomonoenols (**Figure 1**; Pellaud et al., 2018). The core tocochromanol biosynthetic pathway has been widely investigated and is now well-characterized (Mène-Saffrané and DellaPenna, 2010; DellaPenna and Mène-Saffrané, 2011; Mène-Saffrané and Pellaud, 2017). In contrast, our knowledge of the biosynthesis and transport of tocochromanol precursors is currently much more limited. This topic is central in understanding and manipulating tocochromanol metabolism as it is now largely accepted that precursor availability is a major mechanism determining both the type and amount of tocochromanols produced by plants (Mène-Saffrané and Pellaud, 2017). This review summarizes both consensual knowledge and divided views on the biosynthesis and metabolic origins of the tocochromanol precursors HGA and polyprenyl pyrophosphates. It notably highlights the current research gaps on chloroplast membrane transporters required for exchanging tocochromanol precursors between the cytoplasm and plastids.

## Biosynthesis and Origin of Homogentisate

**FIGURE 2:**
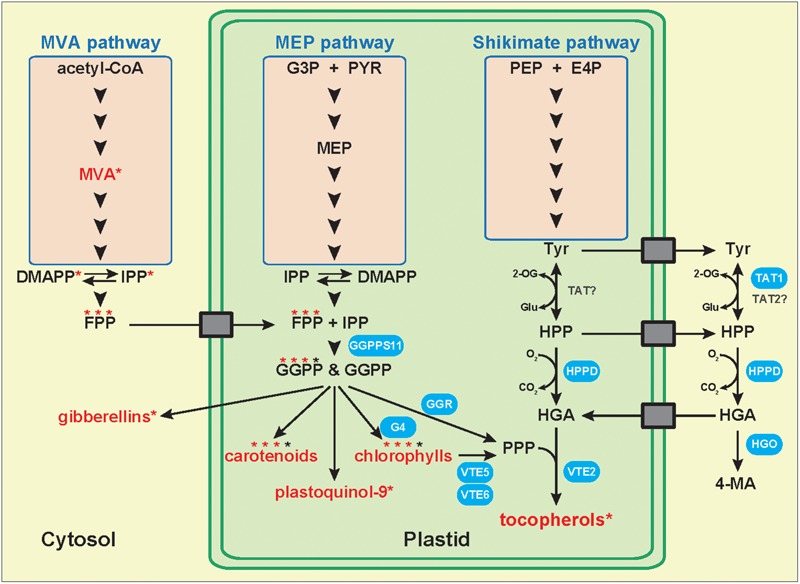
Metabolic origins of tocopherol biosynthetic precursors. Model representing the biosynthetic pathways, cellular compartments, and membrane transport proteins involved in the biosynthesis and transport of tocopherol precursors. Several studies suggest that the cytoplasmic MVA pathway might supply prenyl biosynthetic precursors such as farnesyl pyrophosphate for the synthesis of plastidic isoprenoids including carotenoids, plastoquinol-9, chlorophylls, and tocopherols. Compound names in red with an asterisk indicate that the corresponding radiolabeled molecule has been detected following feeding with radiolabeled mevalonate (cytoplasmic MVA pathway). Compound names topped with three red stars and one black star indicate that three quarters of the prenyl side chain of the molecule was radiolabeled following radiolabeled MVA feeding, while one quarter remained unlabeled. Biosynthetic enzymes in blue indicate that they have been demonstrated to be involved in tocopherol metabolism and that their subcellular localization was confirmed. In contrast, enzyme names in black have not been demonstrated involved in tocochromanol metabolism yet. Genetic and biochemical evidence in Arabidopsis and soybean show that HGA synthesis is localized in the cytoplasm. This implies the existence of membrane transport proteins exporting tyrosine and potentially HPP from plastids to the cytoplasm, and of transporter(s) importing HGA back into plastids. None of the transporters symbolized by gray boxes have been identified yet. CoA, coenzyme A; DMAPP, dimethylallyl pyrophosphate; E4P, D-erythrose-4-phosphate; FPP, farnesyl pyrophosphate; G3P, glyceraldehyde-3-phosphate; G4, chlorophyll synthase; GGPP, geranylgeranyl pyrophosphate; GGPPS, geranylgeranyl pyrophosphate synthase; GGR, geranylgeranyl reductase; Glu, L-glutamate; HGA, homogentisic acid; HGO, homogentisic acid dioxygenase; HPP, 4-hydroxyphenylpyruvate; HPPD, 4-hydroxyphenylpyruvate dioxygenase; IPP, isopentenyl pyrophosphate; 4-MA, 4-maleylacetoacetate; MEP, methylerythritol phosphate; MVA, mevalonate; 2-OG, 2-oxoglutarate; PEP, phosphoenolpyruvate; PPP, phytyl pyrophosphate; PYR, pyruvate; TAT, tyrosine aminotransferase; Tyr, tyrosine; VTE, vitamin E biosynthetic enzyme.

Homogentisate is an aromatic compound resulting from the degradation of L-tyrosine (tyr; **Figure 2**). Following its synthesis by the plastidic shikimate pathway, tyr is converted into 4-hydroxyphenylpruvate (HPP) by tyrosine aminotransferases (TATs), a class of enzymes that catalyze the transamination between tyr and 2-oxoglutarate, and HPP and L-glutamate (Maeda and Dudareva, 2012). Based on sequence similarities, 6–10 *TAT* genes have been identified in the Arabidopsis genome (Riewe et al., 2012; Wang et al., 2016). However, the enzymatic activity has been experimentally confirmed only for *AtTAT1* and *AtTAT2* (Prabhu and Hudson, 2010; Grossmann et al., 2012; Wang et al., 2016). AtTAT1 (also named TAT7) controls 35–50% of the leaf tocopherol biosynthesis (Riewe et al., 2012). The other TAT(s) involved in the biosynthesis of the 50–65% TAT1-independent tocochromanols remain to be identified. They might include the cytosolic AtTAT2 and/or plastidic TAT(s) whose activity has been detected but the corresponding gene(s) has not yet been identified (Wang et al., 2016). It was recently shown that AtTAT1 is localized in the cytoplasm, indicating that HGA synthesis occurs, in total or in part, in this compartment (Wang et al., 2016). This data implies that tocochromanol synthesis involves a yet unknown transporter that brings cytoplasmic HGA back into plastids (**Figure 2**). The TAT isoform(s) involved in seed tocochromanol synthesis remains to be identified.

Hydroxyphenylpruvate is converted into HGA by the 4-hydroxyphenylpyruvate dioxygenase (HPPD), an Fe(II)-containing non-heme oxygenase encoded by a single gene in Arabidopsis (Norris et al., 1995; Tsegaye et al., 2002). Several studies investigating the subcellular localization of HPPD showed that it varies according to the plant species. Early work on *Spinacia oleracea* and *Lemna gibba* localized the HPPD activity in chloroplasts (Löffelhardt and Kindl, 1979; Fiedler et al., 1982). In tomato and cotton, *HPPD* sequence analyses identified a chloroplast transit peptide (Moshiri et al., 2007). In maize, transient expression of the full length *HPPD* gene localized the protein in chloroplasts (Siehl et al., 2014). In contrast, HPPD activity and/or protein is localized in the cytoplasm of both carrot and Arabidopsis cells (Garcia et al., 1997; Wang et al., 2016). Besides these species, soybean HPPD was demonstrated to be localized in both compartments. The single copy *GmHPPD* gene exhibits two transcription start sites that produce a long and a short polypeptide, respectively. Transient and stable expression of both transcripts confirmed that the long version was imported in chloroplasts while the shorter one remained in the cytoplasm (Siehl et al., 2014). The recent identification of the soybean MO12 mutant further supports the cytoplasmic synthesis of the tocochromanol precursor HGA in some plant species. This mutant, which carries a defective *HOMOGENTISATE-1,2-DIOXYGENASE 1* (*HGO1*) gene degrading HGA into 4-maleyl acetoacetate, overaccumulated free HGA and tocochromanols in seeds (Stacey et al., 2016). Interestingly, *GmHGO1* sequence analysis did not detect any obvious targeting peptide suggesting that HGO1 is likely localized in the cytoplasm. This was experimentally confirmed with transient expression of the HGO1:GFP fusion protein that accumulated exclusively in the cytoplasm. Since the cytoplasmic HGA catabolism directly impacts tocochromanol accumulation in soybean seeds, this implies that cytoplasmic HGA substantially contributes to tocochromanol synthesis, at least in soybean (**Figure 2**). These data further support the existence of a mechanism transferring cytoplasmic HGA into plastids where it is notably used for tocochromanol synthesis. To date, the identity of this membrane transporter(s) remains to be identified. Interestingly, the Arabidopsis *HGO* gene is also predicted to be localized in the cytoplasm (TAIR10).

## Biosynthesis of the Tocochromanol Isoprenoid Side Chains

All four polyprenyl tocochromanol precursors (PPP, GGPP, SPP, and THGGPP) derive from GGPP produced by GERANYLGERANYL PYROPHOSPHATE SYNTHASEs (GGPPS; **Figure 2**). Among the 10 GGPPS identified in Arabidopsis, seven are predicted to be localized in plastids, notably GGPPS11 that is the main paralog responsible for the synthesis of GGPP used for tocochromanol production (Ruiz-Sola et al., 2016).

The prenyl precursor PPP used for tocopherol synthesis mostly comes from chlorophyll catabolism during which its side chain is cleaved off and recycled (Valentin et al., 2006; Vom Dorp et al., 2015). To date, four hydrolases active on chlorophylls and/or on their Mg-free derivative pheophytines have been identified. Two *CHLOROPHYLLASEs AtCLH1* and *AtCLH2* genes have been isolated in Arabidopsis based on sequence homologies with the *Citrus sinensis* and *Chenopodium album CLH* genes, respectively (Jacob-Wilk et al., 1999; Tsuchiya et al., 1999). The chlorophyllase activities of CsCLH, CaCLH, and AtCLH1 have been demonstrated *in vitro*. Intriguingly, neither *AtCLH1* nor *AtCLH2* genes exhibit the typical chloroplast transit peptide targeting them into plastids where chlorophyll catabolism occurs. In addition, both AtCLH1 and AtCLH2 have been shown to have a very limited role in chlorophyll catabolism occurring during senescence (Schelbert et al., 2009). The hydrolase responsible for chlorophyll dephytylation during leaf senescence is encoded by the *PHEOPHYTINASE* gene (Schelbert et al., 2009). This enzyme is localized in plastids and is specifically expressed during senescence. Consequently, leaves of *pph* mutants exhibit a stay-green phenotype and accumulate pheophytin upon dark-induced senescence. Although it has not been experimentally tested yet, it is very likely that the tocopherol accumulation observed in senescent leaves is dependent on this enzyme (Rise et al., 1989). Recently, it was shown that the chlorophyll dephytylase CLD1 catalyzes the dephytylation of chlorophylls both *in vitro* and *in vivo* (Lin et al., 2016). The *CLD1* gene is strongly expressed in green leaves where the corresponding protein is associated with thylakoids in plastids. As for chlorophyll catabolism in Arabidopsis seeds, it has been shown that PPH, CLH1, and CLH2 are not involved in supplying phytol for seed tocopherol synthesis (Zhang et al., 2014). The enzyme catalyzing this step in seeds of chloroembryophytes is currently unknown.

Once cleaved from chlorophyll/pheophytin, phytol needs to be phosphorylated twice to produce PPP. This reaction is catalyzed by the phytol kinase VTE5 and the phytyl phosphate kinase VTE6 (**Figure 2**). Seed and leaf tocopherols of *vte5* mutants are reduced by 80 and 65%, respectively, while *vte6* leaves fully lack tocopherols (Valentin et al., 2006; Vom Dorp et al., 2015). Since phytol content was significantly increased in *vte6* mutants, it has been suggested that VTE6 might also directly phosphorylate phytol. Thus, according to this later biosynthetic model, tocopherol synthesis would fully rely on chlorophyll catabolism and recycling. This is only partially supported by data obtained with mutants of the chlorophyll synthase *G4/CHLSYN1*, the enzyme catalyzing the last step of chlorophyll biosynthesis. Indeed, while leaves of *g4/chlsyn1* mutants maintained for 5 weeks on 2% sucrose were nearly devoid of tocopherols, their seeds still accumulated 25% wild-type tocopherols (Zhang et al., 2015). These results support the idea that leaf tocopherol synthesis is fully dependent on chlorophyll catabolism and phytol recycling. In contrast, they suggest that seed tocopherol synthesis might partially depend on a source of polyprenyl other than chlorophylls that has not been identified yet.

The PC-8 prenyl precursor SPP is produced by the plastidic SOLANESYL PYROPHOSPHATE SYNTHASEs (SPS) 1 and 2 (Block et al., 2013). *In vitro* experiments with recombinant AtSPS1 and 2 and various polyprenyl pyrophosphates in combination to either isopentenyl pyrophosphate (IPP) or dimethylallyl pyrophosphate (DMAPP) showed that both enzymes poorly utilize geranyl pyrophosphate and DMAPP (Hirooka et al., 2003, 2005). In contrast, both efficiently utilize farnesyl pyrophosphate and GGPP in combination to IPP with the highest affinity for GGPP/IPP. Double *sps1 sps2* mutants are albino and lack plastoquinol-9, the prenylated benzoquinol notably involved in the electron transfer chain of photosynthesis, and PC-8, the tocochromanol resulting from plastoquinol-9 cyclization.

The tocomonoenol prenyl precursor THGGPP is an intermediate of the reductive pathway converting GGPP into PPP (Kruk et al., 2011; Pellaud et al., 2018). This process is mediated by a geranylgeranyl reductase (GGR) that sequentially converts both geranylgeranylated chlorophyll *a* and GGPP into phytylated chlorophyll *a* and PPP, respectively (Keller et al., 1998; Takahashi et al., 2014). This biosynthetic model originates from biochemical experiments in which both dihydro- and tetrahydrogeranylgeranyl intermediates were identified together with phytylated chlorophyll and PPP following the incubation of geranylgeranyl chlorophyll *a* or GGPP with recombinant GGR, respectively (Keller et al., 1998). Recently, the sequential reduction of geranylgeranyl intermediates has been further confirmed *in planta* with the functional study of LIGHT-HARVESTING CHLOROPHYLL-BINDING-LIKE (LIL) 3:1 and 3:2 that both stabilize GGR (Takahashi et al., 2014). Mutations in either *LIL3:1* or *LIL3:2* lead to the reduction of GGR activity and to the concomitant accumulation of dihydro- and tetrahydrogeranylgeranyl chlorophylls in leaves. Moreover, leaves of *lil3:1 lil3:2* double mutants exhibit further lower GGR activity and mostly accumulate geranylgeranylated chlorophylls. Since tocomonoenols were not identified in Arabidopsis seeds at the time of that study, which focused exclusively on leaves, it is currently unknown whether tocomonoenol contents increase in seeds of *lil3:1* and *lil3:2* single mutants. In line with this tocomonoenol biosynthetic model, we recently showed that γ-tocomonoenol content significantly increased in segregating *GGR ggr* seeds (Pellaud et al., 2018).

## Metabolic Origins of the Tocochromanol Isoprenoid Side Chains

Most articles on plant tocochromanol metabolism attribute the origin of the prenyl component of tocochromanols exclusively to the plastidic methylerythritol phosphate (MEP) pathway. These references cite two significant review articles published right after the discovery of this non-mevalonate isoprenoid pathway that did not support the exchange of isoprenoid precursor(s) between the plastidic MEP and cytoplasmic mevalonate (MVA) pathways at that time (Lichtenthaler, 1998, 1999). Recently, another landmark review article on the MVA and MEP pathways also supported the MEP pathway origin of the prenyl component of plastidic isoprenoids by using two lines of genetic evidences (Vranová et al., 2013). Germinating homozygous mutants of MEP biosynthetic genes are albino indicating that the MVA pathway is not capable of complementing chlorophyll synthesis during germination of MEP pathway mutants. In addition, mutants of MVA biosynthetic genes are male sterile indicating that the MEP pathway is not capable of complementing the defective pollen of MVA pathway mutants. Besides these genetic data, much evidence shows that the prenyl component of plastidic isoprenoids might also come from the cytosolic MVA pathway. Independent feeding experiments using radiolabeled MVA, an intermediate of the cytoplasmic MVA pathway, resulted in the production of radiolabeled isoprenoids typically produced in plastids. It was shown for instance that maize shoots, calendula leaves, and barley leaves treated with [2-^14^C]MVA produced radiolabeled α-tocopherol (Threlfall et al., 1967; Janiszowska et al., 1976; Schultz, 1990). This suggests that cytoplasmic MVA pathway-derived IPP/DMAPP and/or prenyl pyrophosphates might be imported into plastids and incorporated into plastidic isoprenoids such as tocopherols. Similarly, treatment of pine seedlings, etiolated maize and oat seedlings, potato sprouts, and barley leaves with [2-^14^C]MVA resulted in the synthesis of chlorophylls with labeled phytyl side chains (Treharne et al., 1966; Wieckowski and Goodwin, 1967; Schultz, 1990; Kozukue et al., 2001). These results were recently confirmed in cotton leaves in which up to 19 and 44% of the phytyl side chains of chlorophyll *a* and *b*, respectively, were labeled following [2-^13^C]MVA feeding (Opitz et al., 2014). Since the phytyl side chain of chlorophylls is a significant prenyl source for tocopherol synthesis, these data further indicate that the prenyl component of tocopherols likely does not originate exclusively from the plastidic MEP pathway and might also originate from the cytoplasmic MVA pathway. Moreover, labeled β-carotene has been independently detected in maize and barley shoots, in etiolated maize and oat seedlings, in pine seedlings, in barley leaves, and in cultured cells of liverworts treated with [2-^14^C]MVA (Treharne et al., 1966; Threlfall et al., 1967; Wieckowski and Goodwin, 1967; Schultz, 1990; Nabeta et al., 1997). A quantitative study recently estimated that 34% of β-carotene in cotton leaves was labeled following [2-^13^C]MVA feeding (Opitz et al., 2014). While these feeding studies do not firmly demonstrate the existence of a systematic cross-flow between the cytoplasmic and plastidic isoprenoid pathways, they undoubtedly demonstrate that cytoplasmic isoprenoid precursor(s), when available in large amount, are incorporated into plastidic isoprenoids such as tocopherols, carotenoids, and chlorophylls. These data also indicate that a transport system allowing the import of cytoplasmic isoprenoid precursor(s) into plastids exists in plants (**Figure 2**). To date, this transporter(s) remains to be identified.

Recent feeding experiments with radiolabeled MVA combined to NMR analysis further refined our understanding of the import of cytoplasmic MVA pathway-derived isoprenoid precursors into plastids. It was independently shown that three quarters of the prenyl units composing the phytyl side chain of chlorophylls, β-carotene or other plastidic isoprenoids produced after [2-^13^C]MVA feeding were labeled whereas the terminal C5 unit was not (Itoh et al., 2000; Karunagoda et al., 2001; **Figure 2**). This suggests that MVA pathway-derived radiolabeled IPP is concatenated in the cytoplasm into radiolabeled FPP that is imported into plastids where it is condensed with non-labeled MEP pathway-derived IPP. This biosynthetic model is supported by data showing the import of cytosolic FPP into plastids and its incorporation into the phytyl side chain of chlorophylls and into β-carotene (Nabeta et al., 1995, 1997). Moreover, comparison of plastid uptakes of several radiolabeled polyprenyl pyrophosphates showed that FPP was 6–8 and 2–5 times more incorporated into the side chains of chlorophylls than IPP or GPP, respectively (Karunagoda and Nabeta, 2004). Collectively, these data indicate that FPP is likely the preferential cytoplasmic isoprenoid precursor imported into plastids and used for the synthesis of plastidic isoprenoids (**Figure 2**).

Several metabolic engineering studies overexpressing cytoplasmic isoprenoid biosynthetic genes further support the utilization of cytoplasmic MVA-derived isoprenoid precursors for the synthesis of plastidic isoprenoids. It was shown for instance that overexpression of *Brassica juncea 3-HYDROXY-3-METHYLGLUTARYL-COA SYNTHASE1*, an MVA pathway biosynthetic gene, did not only increase the accumulation of cytoplasmic phytosterols in transgenic tomato fruits, but also strongly increased the synthesis of plastidic isoprenoids such as α-tocopherol (fivefold) and carotenoids (twofold; Liao et al., 2017). Overexpression of a *Salvia miltiorrhiza* 3-HYDROXY-3-METHYLGLUTARYL-COA REDUCTASE, a rate-limiting enzyme of the MVA pathway, strongly increased the synthesis of tanshinone, a diterpene produced by the MEP pathway (Kai et al., 2011). Similarly, overexpression in spike lavender of the Arabidopsis 3-HYDROXY-3-METHYLGLUTARYL-COA REDUCTASE1, an MVA pathway biosynthetic gene, doubled the synthesis of monoterpenes produced by the MEP pathway (Muñoz-Bertomeu et al., 2007). Together with the feeding experiment data presented above, these metabolic engineering studies further support the concept that MVA-derived isoprenoid precursors are used for the synthesis of plastidic isoprenoid compounds when they are available in sufficient amounts. This conclusion might explain why the MVA pathway does not complement chlorophyll synthesis in albino seedlings of MEP pathway mutants, and why the MEP pathway does not complement the defective pollen of MVA pathway mutants. Indeed, analysis of MEP and MVA biosynthetic gene expression with ePlant (The Bio-Analytic Resources for Plant Biology; University of Toronto) shows that MVA biosynthetic genes are very weakly expressed in seedlings, notably the mevalonate kinase encoded by a single gene (At5g27450). Similarly, MEP biosynthetic genes are very weakly expressed in pollen, notably the 1-deoxy-D-xylulose-5-phosphate reductoisomerase also encoded by a single gene (At5g62790). Thus, if both pathways are not fully functional at a given physiological stage, they cannot complement the one disrupted by the mutation, and this, despite the possible exchange of isoprenoid precursor(s) showed by feeding experiments and metabolic engineering. Importantly, there is currently no report in the literature demonstrating the dual origin of the prenyl side chain of tocochromanols in wild-type plants.

## Conclusions and Perspectives

From the cloning of the first tocopherol biosynthetic genes to the recent identification of the tocomonoenol ones, substantial progress has been made in understanding the core tocochromanol biosynthetic pathways in plants (Shintani and DellaPenna, 1998; Pellaud et al., 2018). In contrast, major knowledge gaps highlighted in the present review currently exist on the metabolic origin(s) of tocochromanol biosynthetic precursors and on transporters involved in exchanges of polar and lipophilic metabolites between the cytoplasm and plastids. For instance, the cytoplasmic biosynthesis of HGA notably implies the existence of membrane transport proteins exporting tyr and potentially HPP from plastids to the cytoplasm, and of transporters importing back HGA into plastids. Since HGA availability is a potent mechanism controlling tocochromanol synthesis in plants, future challenges will be to identify these transporters and modulate their expression to investigate their role in tocochromanol biosynthesis. Similarly, although the existence of mechanisms importing cytoplasmic MVA-derived isoprenoid precursors into plastids is no longer to be demonstrated, these transporters remain to be identified and characterized. In addition, since the incorporation of MVA pathway-derived prenyls into tocochromanols has not been demonstrated yet in wild-type plants, it is still an open question whether the cytoplasmic MVA pathway contributes or not to tocochromanol biosynthesis in plants.

## Author Contributions

SP and LM-S designed and wrote the manuscript.

## Conflict of Interest Statement

The authors declare that the research was conducted in the absence of any commercial or financial relationships that could be construed as a potential conflict of interest.
